# Working towards risk stratification for ascending aortic dilatation in pediatric Turner syndrome patients: results of a longitudinal echocardiographical observation

**DOI:** 10.1007/s00431-023-05344-y

**Published:** 2023-11-27

**Authors:** J. Heno, I. Michel-Behnke, C. Pees

**Affiliations:** Department of Pediatric Cardiology, Pediatric Heart Center Vienna, University Hospital for Children and Adolescent Medicine, Medical University Vienna, Waehringer Guertel 18-20, A – 1090 Vienna/Wien, Austria

**Keywords:** Aortic dilatation, Bicuspid aortic valve, Pediatric cardiology, Turner syndrome

## Abstract

This study aimed to longitudinally evaluate aortic root dimensions and elasticity in pediatric Turner syndrome (TS) in relation to known cardiac implications such as coarctation of the aorta (CoA) and bicuspid aortic valves (BAV) in order to create an improved risk profile for the presumed underlying vessel pathology in childhood. We report on the longitudinal findings of our pediatric TS outpatient clinic over a period of up to 7.6 years. Forty-nine TS patients (median age at baseline 9.7 ± 5.9 years, range 0–19.8) were followed-up for on average 2.9 ± 1.1 examinations and a median time of 3.4 ± 1.6 years. Aortic root (AoR) diameters and corresponding *Z*-scores were determined echocardiographically, and elasticity parameters as well as annual progression rates were calculated. At baseline, 16.3% of patients showed *Z*-scores > 2 at one or more levels of the AoR (35.7% of patients with BAV, odds ratio of 4.2). There was net progression to be noted at all measuring levels, leading to 28.6% of patients (50% of patients with BAV) exhibiting aortic dilatation at the end of follow-up. Progression correlated with the presence of BAV, non-mosaic monosomy, and age. A levelling-off of progression was seen with the onset of adolescence.

*Conclusions*: Marked progression of aortic diameters leading to the development of dilatation can be observed in TS patients during childhood and stresses the importance of close surveillance during childhood. Main risk factors are BAV and complete monosomy 45X0. A beneficial influence of estrogen substitution can be suspected but needs further investigation.

**Table:** 

**What is known:** *• Patients with Turner syndrome are at an increased risk for aortic dilatation and dissection.* *• The presence of BAV and complete monosomy 45X are additional risk factors.*	
**What is new:** *• Aortic dilatation can be detected in pediatric patients with Turner syndrome.* *• Relevant progression in childhood is possible in at-risk individuals and warrants close surveillance.*

**What is known:**

*• Patients with Turner syndrome are at an increased risk for aortic dilatation and dissection.*

*• The presence of BAV and complete monosomy 45X are additional risk factors.*

**What is new:**

*• Aortic dilatation can be detected in pediatric patients with Turner syndrome.*

*• Relevant progression in childhood is possible in at-risk individuals and warrants close surveillance.*

## Introduction

Affecting up to 1:2000 live-born females, Turner syndrome (TS) is a relatively common genetic disorder [1]. In addition to severe derangements of endocrine functions, congenital heart defects (CHD) occur in about 30% of patients [2] and half of the significantly increased risk of premature death is due to cardiovascular events [3, 4]. Concerning CHD, bicuspid aortic valves (BAV; 30% [5–7]) and aortic coarctation (CoA; 15% [5, 8]) — mostly co-occurring with BAV — are the most frequent forms, with higher risk of ascending aortic dilatation (AoD; 15–45%; [9–13]), starting at a relatively young age [14]. The incidence of aortic dissection (AD) is increased 20- to 100-fold, taking place at a mean age of 35 years and not limited to individuals with previous aortic dilatation [2, 15–17]. The underlying aortopathy in TS seems not to be restricted to dilated aortic segments, as implied by pathological elasticity changes of the ascending aorta, which are present as early as in childhood and adolescence [14].

In 2017, Gravholt et al. published clinical guidelines for the care of girls and women with TS [18]. Medical treatment to reduce aortic diameter progression is suggested in girls ≤ 16 years of age with an aortic *Z*-score of ≥ 3 as well as in women > 16 years of age with an indexed aortic diameter ≥ 2.3 cm/m^2^ or if rapid progression of more than 5 mm or 1 *Z*-score is observed over a one-year period. Z-scores were based on the TS indices by Quezada et al. [19]. The indices were extrapolated from a study cohort of 360 TS patients without BAV with an age range of 3–70 years and normalized to body surface area (BSA) and not to age and therefore did not account for changes due to aging, and especially not for the specific subgroup of children and adolescents in particular.

Addressing this, we aimed to analyze aortic dimensions and their annual progression rates in our pediatric TS patient population to better understand the development of aortopathy in young TS patients during growth and to improve the evidence base for decision-making regarding follow-up intervals as well as treatment indications.

## Methods

### Study design

Since 2013, all our pediatric TS patients of eastern Austria under surveillance and treatment in our endocrinology department have regularly been examined. For all patients registered, phenotypical apparent TS was confirmed by karyogram. Inclusion criteria for this longitudinal echocardiographic evaluation were (1) age between 0 and 20 years at baseline and (2) a minimum of two echocardiographic examinations with a follow-up of a minimum of 1 year. Examinations were included at minimum intervals of 1 year to prevent overrepresentation of severely affected patients. Exclusion criteria were (1) lack of written informed consent from all patients and/or their relatives, (2) incomplete or insufficient examinations, (3) cardiac operations other than correction of CoA, (4) CoA repair less than six months before examination, and (5) other connective tissue disorders such as Marfan, Ehlers-Danlos, or Loeys-Dietz syndrome or inflammatory changes of the aorta such as Takayasu arteritis.

The study protocol was approved by the institutional review board of the Vienna University Hospital committee on human research (approval reference number 1197/2018).

### Echocardiography

All examinations were carried out by the same investigator (CP). The echocardiographic assessment — performed with a Vivid E9 GE Medical device (Horton, Norway) — included measuring diameters of the aortic valve (AV), aortic root (AoR), sinutubular junction (STJ), and ascending aorta (AoA) perpendicular to the aortic flow in the parasternal long axis using the inner-edge to inner-edge method. All values were normalized to the current BSA of the patient, and data is presented as corresponding *Z*-score values according to the dataset by Warren et al. [20]. *Z*-scores ≥ 2 were considered as indicating dilatation. Baseline data of all four aortic measurement levels were as well expressed in the TS specific *Z*-score percentile graphs by Quezada et al. [19]. The elastic properties of the AoR were evaluated by M-mode measurements in parasternal long axis view. Offline calculations of mean systolic and diastolic diameters were performed for the calculation of stiffness and distensibility as previously described [21]. Blood pressure was measured simultaneously on the right upper arm using a Philips SureSignsVS2 (Andover, MA, USA); in patients with CoA, blood pressure was measured at all four limbs.

### Statistical analysis

Data analysis was performed with IBM Statistics, version 26.0 (SPSS, Inc., Chicago). Kolmogorov–Smirnov tests were used to test for parametricity. For continuous variables, Student’s *t*-test, and for nonparametric parameters, Kruskal Wallis/Man-Whitney *U* computations were carried out. Correlation structures were tested using Pearson’s and Spearman’s correlation coefficient, as applicable. Multiple regression analyses were used to quantify the impact of various presumed risk factors. Data is given as two-sided *p*-values, which were considered statistically significant if < 0.05.

## Results

### Baseline characteristics

Forty-nine patients of our outpatient clinic fulfilled the inclusion criteria and all approved their participation in this study. Demographic baseline data is depicted in Table 1, showing a median age at baseline with 9.7 ± 5.9 (range 0–19.8) years with a balanced age distribution with 12 patients ≤ 5 years, 13 patients each between 5 to ≤ 10 years and 10 to ≤ 15 years, and 11 patients > 15 years of age. Patients were followed-up for on average 2.8 ± 1.0 examinations (range 2–6) and a median follow-up time of 3.4 ± 1.5 (range 1.5–7.6) years. While on average, body mass index (BMI) percentiles were within the normal ranges, eight patients were deemed overweight (> 85th percentile) and one obese (> 96th percentile). Blood pressure measurements taken during the echocardiographic examinations showed normal average percentiles. Fourteen patients (28.6%) were diagnosed with BAV but no case of aortic valve stenosis or more than mild aortic regurgitation was noted. Seven of the 14 patients with a BAV presented with additional CoA and one further patient had isolated coarctation (2.0%), adding up to 16.3% of patients with CoA. Seven of the eight patients with CoA were operated on within 10 days after birth. Four patients received an angiotensin converting enzyme inhibitor, three of these four only during the first months after CoA repair.

**Table 1:** Demographic data of the study population in total and subdivided by presence of BAV

**Demographic data**	**Total study population**	**TAV baseline (35/49)**	**BAV baseline (14/49)**	***p*-value (TAV-BAV)**
Age (years) [range]	9.7 ± 5.9 [0–19.8]	10.6 ± 5.2 [0.6–17.8]	7.5 ± 7.1 [0–19.8]	0.097
Time of follow-up (years) [range]	3.4 ± 1.5 [1.5–7.6]	3.3 ± 1.4 [1.9–6.3]	3.6 ± 1.9 [1.5–7.6]	0.757
Number of examinations [range]	2.8 ± 1.0 [2 – 6]	2.5 ± 0.8 [2 – 5]	3.4 ± 1.3 [2 – 6]	0.01
Height (cm)	120.6 ± 32.0	126.9 ± 26.2	104.6 ± 39.8	0.054
Weight (kg)	31.5 ± 18.2	34.0 ± 16.9	25.3 ± 20.3	0.131
BMI percentile	59.5 ± 28.2	62.9 ± 25.0	51.2 ± 34.5	0.266
Systolic BP percentile	73.6 ± 24.9	81.2 ± 23.4	72.3 ± 28.4	0.341
Diastolic BP percentile	73.6 ± 23.9	71.2 ± 22.7	79.7 ± 26.9	0.164
BAV (*n*)	14/49 = 28.6%	0	14	
CoA (*n*)	8/49 = 16.3%	1	7	
Non-mosaic monosomy 45X0 (*n*)	27/49 = 53%	16/35 = 46%	11/14 = 79%	

Values are numbers or means ± SD

*BP* blood pressure, *BSA* body surface area, *BMI* body mass index

The genetic results showed 27 patients (53%) with a complete monosomy 45X; the remaining 22 patients had different forms of mosaicism. Of the 14 patients with BAV, 11 had monosomy 45X, as well as six out of seven patients with combined BAV and CoA.

Patients with BAV did not show significant differences in baseline parameters concerning age, time of follow-up, auxology, or blood pressure measurements compared to the subgroup with tricuspid aortic valves (TAV), but were slightly younger and were examined significantly more frequently (Table 1).

In total, 78% of patients received growth hormone and 61% estrogen therapy. At the onset of growth (GH) and sex hormone replacement therapy (SHRT), patients were on average 6.7 ± 3.2 and 12.7 ± 1.6 years of age, respectively. Patients with complete monosomy were slightly younger at the start of GH replacement therapy compared to those with mosaicism (6.2 ± 3.4 vs. 7.1 ± 2.9 years, n.s.), SHRT was introduced at similar age (complete monosomy 12.8 ± 1.7 vs. mosaicism 12.6 ± 1.6 years, n.s.).

### Aortic diameters

Aortic diameter measurements as well as *Z*-scores of the entire cohort are summarized in Table 2. Eight patients (16.3%) showed a *Z*-score ≥ 2 at baseline at one or more measuring points (AV:0, AoR:5, STJ:4, AoA:6).

**Table 2:** Aortic dimensions and elasticity parameters at baseline and end of follow-up given for the entire study population and subdivided by presence of BAV

**Table II:**	**Total (baseline)**	**Total (end of follow-up)**	***p***	**TAV (35/49) (baseline)**	**BAV (14/49) (baseline)**	***p***	**TAV (end of follow-up)**	**BAV (end of follow-up)**	***p***
**AV** (mm)	15.7 ± 3.9	17.6 ± 3.7	**0.009**	16.1 ± 3.2	14.4 ± 5.2	0.426	18.0 ± 2.8	17.4 ± 4.5	0.644
**AV Z** [range]	0.07 ± 0.95 [− 2– + 1.67]	0.51 ± 1.10 [− 2.2–2.85]	0.092	0.03 ± 0.90 [− 2.0–1.67]	0.19 ± 1.09 [− 1.46–1.62]	0.543	0.39 ± 0.94 [− 2.0–2.93]	0.81 ± 1.42 [-0.84–3.87]	0.301
**AoR** (mm)	21.4 ± 6.0	24.1 ± 4.8	**0.031**	21.9 ± 5.0	20.1 ± 7.9	0.535	24.2 ± 4.2	23.9 ± 6.3	0.874
**AoR Z** [range]	0.32 ± 1.06 [− 1.62– + 2.52]	0.56 ± 1.09 [− 2.2–2.85]	0.267	0.22 ± 0.99 [− 1.44–2.52]	0.58 ± 1.23 [− 1.62–2.47]	0.290	0.41 ± 1.06 [− 2.2–2.39]	0.96 ± 1.11 [− 0.44–2.85]	0.104
**STJ** (mm)	17.3 ± 4.9	19.6 ± 4.1	**0.036**	17.5 ± 4.1	16.6 ± 6.6	0.723	19.2 ± 3.5	20.4 ± 5.6	0.469
**STJ Z** [range]	0.20 ± 1.09 [− 1.88–2.47]	0.43 ± 1.20 [− 2.3–3.39]	0.313	0.045 ± 0.97 [− 1.6–1.7]	0.58 ± 1.30 [− 1.88–2.47]	0.125	0.10 ± 1.04 [− 2.3–2.23]	1.27 ± 1.2 [− 1.24–3.39]	**0.005**
**AoA** (mm)	18.2 ± 5.4	20.1 ± 4.5	0.059	17.9 ± 3.9	19.0 ± 8.2	0.652	19.8 ± 3.7	21.0 ± 6.0	0.49
**AoA Z** [range]	− 0.03 ± 1.74 [− 2.7–4.63]	0.09 ± 1.55 [− 2.5–4.04]	0.637	-0.48 ± 1.27 [− 2.7–3.31]	1.08 ± 2.24 [− 2.43–4.63]	**0.026**	− 0.33 ± 1.28 [− 2.5–2.8]	1.14 ± 1.70 [− 1.73–4.04]	**0.002**
**Stiffness** [range]	4.49 ± 1.75 [2.02–8.66]	4.46 ± 2.25 [1.50–12.34]	0.057	4.49 ± 1.59 [2.28–8.66]	4.49 ± 2.18 [2.02–7.84]	0.53	4.54 ± 2.50 [1.50–12.34]	4.28 ± 1.54 [2.62–6.88]	0.734
**Distensibility** (dyne) [range]	6.27 ± 2.75 [2.67–15.23]	6.45 ± 3.05 [2.03–15.23]	0.092	5.91 ± 2.61 [2.67–15.23]	7.17 ± 3.00 [3.01–11.98]	0.124	6.53 ± 3.34 [2.03–15.23]	6.26 ± 2.25 [3.28–10.42]	0.992
**Distensibility** (kPa) [range]	48.10 ± 20.05 [20.75–99.02]	51.36 ± 24.01 [15.59–125.6]		46.37 ± 17.67 [20.75–90.34]	52.41 ± 25.30 [22.84–99.02]	0.084	51.71 ± 25.98 [15.59–125.58]	50.48 ± 19.04 [25.44–86.3]	0.832

Results considered statistically significant are marked by bold font

*AV* aortic valve annulus, *AoR* aortic root, *STJ* sinotubular junction, *AoA* ascending aorta, *TAV* tricuspid aortic valve, *BAV* bicuspid aortic valve

Comparing the baseline measurements of our 49 patients with the percentile curves of Quezada et al. resulted in equal *Z*-scores at the AV level. Distally, the *Z*-scores according to the Warren data were consistently + 0.5 SD (AoR) and + 1 to + 1.5SD (STJ, AoA) higher.

In regard to this, none of our patients reached a Quezada-*Z*-score level ≥ 3 (i.e., cut-off level for medical treatment) even though there were three patients with a *Z*-score of 3 to 3.5 and one with a *Z*-score of 4.6 according to the Warren data.

Over the entire observation period, all parameters showed a net progression, but these changes per se were not statistically significant.

The fourteen patients with BAV — half of which also had CoA — did not show significant differences in baseline demographics or average length of follow-up compared to those with TAV (Table 1). Baseline aortic diameter *Z*-scores were markedly different especially at the more distal ascending aortic levels; however, significance was only reached at the level of the AoA (Table 2).

At the end of the observation period, in comparison to patients with TAV, *Z*-scores of those with BAV were significantly greater at the level of the STJ and AoA with 0.10 ± 1.04 compared to 1.27 ± 1.20, and −0.33 ± 1.28 compared to 1.14 ± 1.70, with *p* = 0.005 and *p* = 0.002, respectively. At the directly supravalvular measuring levels, median *Z*-scores were approximately +0.5 higher in those with BAV, although this did not show statistical significance. Patients with TAV were most likely to develop aortic dilatation at the level of the AoR, while for patients with BAV, dilatation presented more distally. In total, the TAV group exhibited rather low *Z*-score progressions over the observation period between 0.03 ± 0.26/year (STJ) and 0.16 ± 0.43/year (AV). The BAV group showed relatively similar progression rates, but reaching significantly higher progression rates at the STJ level (0.26 ± 0.35/year). Accordingly, Spearman’s test showed moderate correlation of BAV with aortic dimensions at STJ and AoA levels despite the small subgroup sizes (STJ: *r* = 0.35; AA: *r* = 0.41; both *p* < 0.001).

**Fig. 1:**
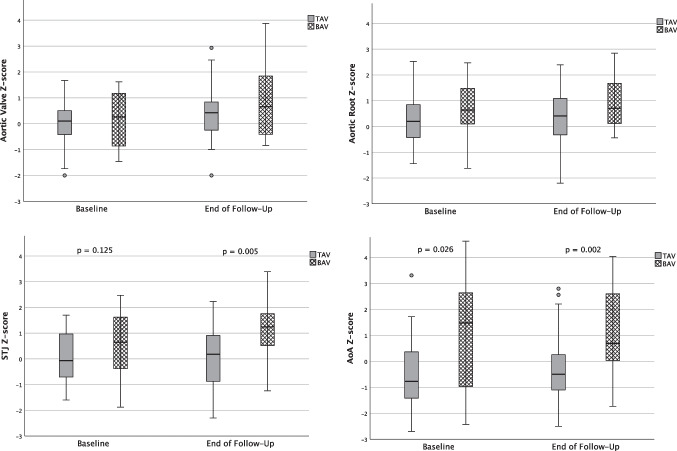
Boxplot depiction comparing baseline indexed aortic diameters with end of follow-up, subdivided by presence of BAV

At the end of follow-up, the number of patients with a *Z*-score of ≥ 2 at any level of the ascending aorta had increased from eight to 14 (28.6%). Three (8.6%) of TAV versus five (35.7%) of BAV patients showed dilatation at baseline compared to seven (20%) of TAV and seven (50%) of BAV at the end of follow-up, with one patient with BAV having an entirely dilated aorta at all four measuring points. This corresponds to a fourfold increased risk of at least one *Z*-score ≥ 2 (odds ratio 4.15) in BAV TS patients (Fig. 1).

At all four measurement levels of the ascending aorta, patients with monosomy 45X tended to show larger aortic diameters at baseline as well as at end of follow-up, however, statistical significance was not reached.

Spearman correlation equations revealed a distinct correlation of age and aortic diameter z-scores (AV *r* = 0.41, *p* < 0.001; AoR: *r* = 0.45, *p* < 0.001; STJ: *r* = 0.39, *p* < 0.001; and AoA: *r* = 0.25; *p* = 0.003). Therefore, we divided our TS population into age subgroups of < 10 and > 10 years of age in order to investigate whether changes can be pinpointed to a certain age range, the start of puberty or the start of estrogen therapy. Both groups were of the same size with *n* = 25 vs. *n* = 24, and the remaining demographic data at baseline did not differ, although length of follow-up was shorter in the older age group. Aortic *Z*-scores showed marked differences at all points of measurement, the younger subgroup showed on average rather smaller aortic diameters compared to the older subgroup with −0.30 ± 1.07 vs. 0.46 ± 0.61 at the AV (*p* = 0.004), −0.12 ± 0.98 vs. 0.78 ± 0.96 at the AoR (*p* = 0.002), −0.33 ± 1.00 vs. 0.75 ± 0.90 at the STJ (*p* < 0.001), and −0.53 ± 1.58 vs. 0.48 ± 1.78 at the AoA (*p* = 0.041). When only including patients with TAV in this sub-analysis, the results were similar. Due to the small subgroup of BAV patients of these different age subgroups, we refrained from a sub-analysis of these patients.

**Fig. 2:**
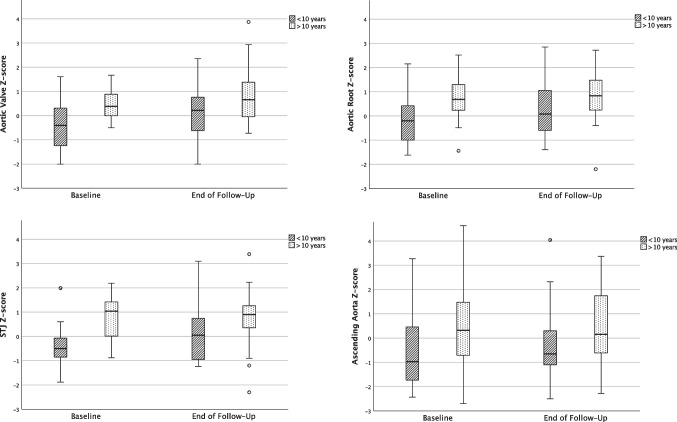
Boxplot depiction comparing indexed aortic diameters at baseline with end of follow-up, subdivided by age group

At the end of follow-up, with the younger group having aged on average 3.8 years, results were still markedly different to the data of the older age group at baseline but did not reach statistical significance with *Z*-scores of 0.2 compared to 0.81 at the level of the AV, 0.29 compared to 0.83 at AoR, −0.16 compared to 0.73 at the STJ, and −0.29SD compared to 0.46SD at the level of the AoA (*p* = 0.054, *p* = 0.088, *p* = 0.096, *p* = 0.092, respectively) (Fig. 2).

**Fig. 3:**
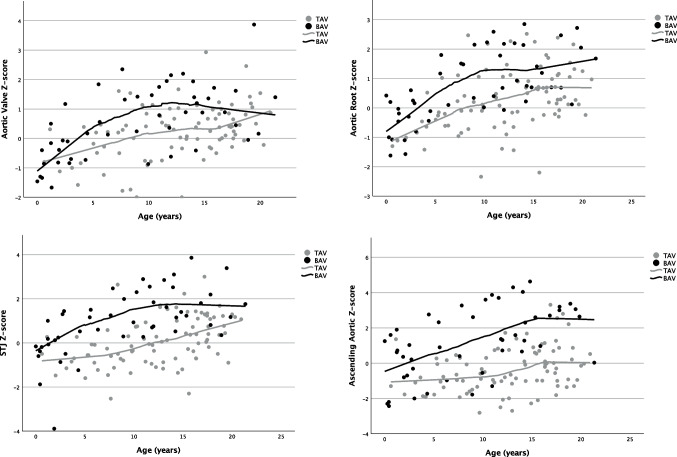
Scatterplot of the development of indexed aortic diameters with age, subdivided by presence of BAV. *Z*-scores according to Warren et al. [20]

In graphical representations, a decelerating increase in *Z*-scores was observed at all measuring points, starting approximately at twelve years of age (Fig. 3).

Aortic elasticity parameters were routinely calculated. With a stiffness index of approximately 4.5, pathological levels were reached both at baseline and end of follow-up (Table 2). Subdivision according to the presence of BAV, age group, and karyotype also showed similar values over the entire observation period. Robust correlation analysis showed that elasticity parameters did not correlate with aortic diameter growth and did not predict absolute aortic size.

### Multivariate regression analysis

Assumed risk factors BAV, COA, age, genotype, and SHRT were integrated by multivariate regression. At all levels of measurement, age was the leading predictor of *Z*-score outcomes (AV adjusted *R*^2^ 21.6%, *p* < 0.001; AoR 29.9%, *p* < 0.001; STJ 30.6%, *p* < 0.001; AoA 11,8%; *p* = 0.016). BAV improved the model significantly at all levels except for the AV (AoR 36%, *p* = 0.040; STJ 40.8%, *p* = 0.007; AoA 43%, *p* < 0.001). The change was most pronounced at the level of the AoA; here, the presence of BAV increased *Z*-scores by 2.7 (*p* < 0.001). Other presumed risk factors did not lead to a significant alteration of the model and SHRT could not be investigated due to age-dependency.

## Discussion

As aortic dilatation and dissection represent the most serious somatic implications of TS, the AV and AoA have been in the spotlight of the cardiologist’s attention in the care of adult patients for some time now [22, 23]. In pediatrics, however, evidence of degenerative vasculopathy has only recently begun to appear in the literature [13, 24]. Our study group was among the first to report significant differences in aortic size and elasticity parameters in children with TS, pointing to the importance of routine cardiological surveillance. We were also able to show that these abnormalities are not limited to those with BAV or CoA [14].

We now present prospective longitudinal data of this sizable study population of 49 girls and young women with TS, who were followed up for a median of 3.4 and up to 7.6 years. Structural heart defects in our study population were of similar prevalence as those previously reported, and evenly distributed in the different age groups.

We found that distinctive aortic dilatation can not only be detected in childhood but there is also significant deterioration of this presumed aortopathy to be observed.

Although dramatic progression of ascending aortic *Z*-scores of the entire study population was not detected, the subgroup of patients who showed dilatation (*Z*-score > 2) at one of the ascending aortic levels nearly doubled over the observation period (16.3 to 28.6%), stressing the importance of close monitoring at this young age.

Risk factor analysis showed a typical distribution of karyotype, proportion of BAV, and CoA in our population compared to the existing literature. The most important risk factor, as already widely established in the adult population, seems to be the bicuspid AV, being responsible for most of the cases with abnormal *Z*-scores [25]. One third of our BAV patients showed dilatation at least at one measuring level at baseline and this fraction increased to about 50% at the end of the observation period. Predilection sites for this subgroup are the STJ and the AoA. In contrast, patients with TAV showed rather stable *Z*-scores. The influence of CoA is difficult to carve out, as BAV and CoA coincide in most cases.

Even after correcting for the presence of BAVs, which are much more common in those with the complete monosomy, patients with a form of mosaicism tend to have a more benign course of progression although statistical significance was not reached. Elasticity has been shown to reach significantly worse levels in our study group of pediatric TS patients compared to a matched control group before [14]. The changes could not be pinpointed to subgroups with specific additional risk factors such as BAV, age, or karyotype. Our longitudinal analysis confirms this observation with similar and stable values across all subgroups without significant progression with age. Including elasticity parameters as predictors did not improve multivariate regression models.

While there was no significant overall progression, the number of more severely affected individuals increased, both in the BAV and TAV subgroups. This suggests that on top of the known risk factors (1) TS in general, (2) presence of BAV, and (3) age, there might be a further protective or predisposing factor leading to a fraction of TS patients showing quicker progression of aortic diameters.

As stated, aortic dimensions correlated strongly with age in general; however, in graphical analysis, a deceleration of *Z*-score increase was noted during the second decade of life, correlating with the usual start of SHRT. Estrogen is known to have beneficial effects on vasculature, and it seems possible that the start of SHRT might have a positive impact on arterial remodeling, bringing dilatation to a halt. It could further be speculated that the individual hormone release constitutes an additional factor in the patient’s risk profile for aortic dilatation.

Estradiol has been proved to have advantageous effects on cardiomyocytes through the increase of endothelial NO synthase and prostacyclin pathways reducing oxidative stress [26]. Furthermore, estradiol is able to reduce fibrosis by modulating myofibroblast response to vessel strain, decreasing matrix metalloprotease (MMP) activity and collagen accumulation [27]. Hemizygosity of Xp, the gene location of tissue inhibitor of metalloprotease 1 (TIMP1), an inhibitor of MMP activity, has recently been proposed to contribute to the development of BAV and aortopathy [28]. Therefore, a positive effect of SHRT is conceivable and has been proposed by other authors [29, 30]. As SHRT is the current standard of care, systematic research of the effect of estrogens on aortic properties in TS does not seem feasible and has to be substituted by close longitudinal observation of changes in vascular phenotype after introduction of estrogen substitution. Further, multicentric analyses of mosaicisms with specific impact on endogenous sex hormone production (e.g., Xp deletion) in relation to aortic properties could shed light on possible causality.

The study at hand is limited by small subgroup sizes, making evaluation of certain proposed risk factors for vasculopathy difficult. Data was indexed using the Warren et al. data [20], which is not disease-specific, as the authors judge the disease-specific *Z*-score data by Quezada et al. [19] to be based on too small a sample size for use in pediatrics (i.e., BSA < 1 m^2^). The influence of antihypertensive medication as well as CoA repair could also not be assessed for the same reasons. The influence of SHRT could not be investigated as it is the standard of care for all patients with TS. Last, a few less affected patients had longer than annual check-up intervals and thus may be underrepresented in this study.

In conclusion, even in a study population entirely consisting of children and adolescents, the progressive character of TS aortopathy is evident in our longitudinal investigation, especially in non-moisaic karyotypes and, in relation to this, in patients with BAV. This progression seems to be ameliorated by the start of SHRT. Routine cardiac and especially echocardiographical check-ups by a pediatric cardiologist starting at birth and continuing during childhood and adolescence are warranted and should be tailored to the presence of the individual risk profile.

## Data Availability

The datasets generated during and/or analyzed during the current study are available from the corresponding author on reasonable request

## Abbreviationsx

AD: Aortic dissection
AoD: Aortic dilatation
AoR: Aortic root
AV: Aortic valve
AoA: Ascending aorta
BAV: Bicuspid aortic valve
BMI: Body mass index
BSA: Body surface area
CoA: Aortic coarctation
CHD: Congenital heart defect
GH: Growth hormone
GHRT: Growth hormone replacement therapy
MMP: Matrix metalloprotease
SHRT: Sex hormone replacement therapy
STJ: Sinutubular junction
TAV: Tricuspid aortic valve
TIMP1: Tissue inhibitor of metalloprotease 1
TS: Turner syndrome
